# Late Professor P N Saxena: A teacher of excellence

**DOI:** 10.4103/0976-500X.72355

**Published:** 2010

**Authors:** Syed Ziaur Rahman, Rahat Ali Khan

**Affiliations:** *Department of Pharmacology, Jawaharlal Nehru Medical College, Aligarh Muslim University, Aligarh - 202 002, India*

Professor Prem Narain Saxena was born on October 15, 1925 in a respected family of a village Bithrichainpur of Bareilly. It was a joint orthodox family village. From village, he rose to a peak of excellence in medical science career.

Dr. Saxena was the first child of his parents Mr. Raghunandan Prasad and Mrs. Saraswati Devi, apart from three other sisters. He married Ms Rajeshwari Saxena, MA (LT), a working lady but latterly left the job and remained as housewife.

## EDUCATION

Dr. P. N. Saxena took early education first in his hometown village Bithrichainpur. He then shifted to Dehradun at an early age of 6 to 7 years to take admission in the first standard, as his father was posted already in Dehradun as ‘Accountant’ in the Department of Irrigation. Dr. Saxena was a very brilliant student since school days despite of malnutrition, rickets and typhoid. He topped with first position both in matriculation from AP Mission School and Intermediate from DAV College of Dehradun. In the very first attempt, he qualified medical entrance and did MBBS with 10th position from the King George Medical College, Lucknow (now Chattrapati Sahuji Maharaj Medical University) in 1947. He was keen in medical research and hence after getting MD degree in Medicine in 1950, he again did MD in Pharmacology in 1952 from the King George Medical College, Lucknow. In addition, he completed his PhD from the Patna University, Patna, in 1963 and DSc from the Aligarh Muslim University, Aligarh, in 1978.[1]

## CAREER

He joined the Department of Pharmacology of Jawaharlal Nehru Medical College, Aligarh Muslim University, Aligarh, on April 14, 1964 as Professor and Founder Chairman, Department of Pharmacology. He served the Department as Head till September 30, 1985 and in view of his outstanding merits, he was reemployed by the University for two years, that is, till September 30, 1987. Soon after retirement, he was selected as emeritus scientist of ICMR, New Delhi, for a period from September 1, 1987 till September 14, 1990. On January 31, 1991, the University appointed him as ‘Professor Emeritus.’ He was among the few scientists in the university who was offered this ‘Professor Emeritus.’

## CONTRIBUTION, AWARDS AND ACHIEVEMENTS

He is nationally and internationally known as a leading Neuropharmacologist as he worked mostly in the field of neuroscience. He was awarded Fellowship of the Rockefeller Foundation Fellow in USA during 1960-61; Commonwealth Medical Fellowship and then Wellcome Research Fellowship in England during 1970s. In London at the National Institute for Medical Research, he worked on mechanism of action of Pyrogen and in the field of thermoregulation with Wilhelm Feldberg (1900 – 1993),[2] a renowned German-British-Jewish pharmacologist and biologist.

Professor P. N. Saxena has to his credit, approximately 145 published research papers. He wrote Hospital Formulary in 1969 and a book-cum-manual for practical pharmacy and experimental pharmacology laboratory. After serving the university as professor and head of the department of pharmacology, he was then retired from active service on September 30, 1987. In the university, he also worked as Dean, Faculty of Medicine, In-charge, Animal House and on few occasions served as acting Principal of Ajmal Khan Tibbiya College and acting Vice Chancellor of the University. However, despite of his retirement from active service, he continued his research activities as Professor Emeritus in the Department of Pharmacology till late December 1994. During those days, his wife was also suffering from cancer and hence could not manage to visit the department regularly and finally discontinued all his academic services in 1995. She died on March 19, 1996.

He had been the founding member of many academic bodies such as Indian Pharmacological Society, Association of Physiologists and Pharmacologists of India, Indian Medical Association, Indian Academy of Neurosciences and Indian Association for the Advancement of Medical Education in India. Indian National Science Academy (INSA) elected him as Fellow (FNA) in 1989.

Dr. Saxena was very simple, kindhearted and pious person. He used laboratory animals (dogs, rabbit, frog, mice) very gently and was very kind towards all the staff of the medical college.

## LEGACY, FRIENDS AND FAMILY

Dr. P. N. Saxena succumbed to death due to severe second stroke on November 29, 1999. He was admitted to KK Hospital, Aligarh before death. He was fond of Urdu and Persian poetry and also maintained a diary, where he used to write his favourite couplets of Urdu poetry. His mother tongue was Urdu. He had a good friends’ circle in academics. To name a few, Professors R. D. Myers, C. von Euler, W. Feldberg, W. J. Lang, A. Herxheimer, Richard J. Schain, K. P. Bhargava, M. B. Waller, Hakim Syed Zillur Rahman, W. R. Martin, Eleanor Zaimis, J. N. Sinha, B. N. Dhawan were the most closest colleagues.

Coincidentally, both of his parents Mr. Raghunandan Prasad and Mrs. Saraswati Devi died in Aligarh due to paralytic attack. Dr. Saxena invited them in Aligarh to stay with his family and children. Dr. Saxena had two daughters and one son.

## NOTE

Wilhelm Feldberg assisted many research workers who came to England as a part of their Commonwealth Medical Fellowship and Wellcome Research Fellowship. Under this Fellowships, Professor P. N. Saxena, during 1970s, got many papers published together with Wilhelm Feldberg while their vocation at National Institute for Medical Research, Mill Hill, London. Most of the reprints of their research papers are preserved in the Library of Ibn Sina Academy of Medieval Medicine and Sciences, Aligarh, India.[3]
